# Severe Seronegative Myasthenia Gravis Revealed by Rapidly Progressive Dysphagia

**DOI:** 10.7759/cureus.93839

**Published:** 2025-10-04

**Authors:** Christopher Ramos Huamancondor, Maroussia Bronchain, Jonathan Petit

**Affiliations:** 1 Emergency Department, Cliniques Universitaires Saint-Luc (CUSL), Brussels, BEL; 2 Department of Neurosciences, Grand Hôpital de Charleroi (GHDC), Charleroi, BEL; 3 Emergency Department, Grand Hôpital de Charleroi (GHDC), Charleroi, BEL

**Keywords:** clarithromycin, myasthenia gravis crisis, plasmapheresis, progressive dysphagia, respiratory distress, seronegative myasthenia gravis

## Abstract

Dysphagia as the initial symptom of a severe myasthenic crisis is an atypical but well-documented presentation in the literature. A woman in her 40s presented to the emergency department with rapidly progressive dysphagia, initially misdiagnosed as gastro-oesophageal reflux. Two weeks earlier, she had completed eradication therapy for *Helicobacter pylori*. Within 24 hours of admission, she developed bulbar symptoms, dyspnoea, proximal muscle weakness, diplopia, and ptosis. Electromyography confirmed a neuromuscular junction disorder consistent with a myasthenic syndrome. Despite initiation of pyridostigmine, her condition deteriorated, requiring orotracheal intubation and intensive care. She received plasmapheresis, intravenous immunoglobulins, corticosteroids, and mycophenolate mofetil, with significant clinical improvement. The final diagnosis was severe, acute, seronegative myasthenia gravis without any clearly evident etiologic factor. This case highlights the importance of considering myasthenic crisis in the differential diagnosis of atypical dysphagia and acute respiratory failure with neurological signs, particularly in the emergency setting.

## Introduction

Dysphagia as the initial symptom of a severe myasthenic crisis is an atypical but well-documented presentation in the literature [1-4]. Establishing the first diagnosis in the emergency department can be challenging, particularly when the presenting symptoms are initially nonspecific. However, it is crucial to consider serious underlying conditions early, as clinical deterioration may occur rapidly [5]. Myasthenia gravis is a rare autoimmune disease that affects the neuromuscular junction and manifests as muscle weakness [5-7]. A variety of stressors can exacerbate myasthenia, with infection also recognised as the most common precipitating factor [5,8].

Myasthenic crisis is an acute and severe complication of myasthenia gravis, defined by profound muscular weakness that threatens respiratory function and often requires mechanical or non-invasive ventilation [5,6]. It occurs in approximately 15%-20% of patients with myasthenia gravis, typically within the first few years of disease onset [5]. Respiratory tract infections are the most frequently identified triggers [5,8]. However, no specific cause is found in 30%-40% of cases [5]. Patients at increased risk include those with thymoma, anti-muscle-specific kinase (MuSK) antibodies, a previous history of myasthenic crisis, or significant bulbar involvement [5-6,9]. We describe here a case of seronegative myasthenic crisis as a first presentation in the emergency department, atypical due to the rapidity and severity of its onset.

## Case presentation

A 49-year-old woman initially presented to the emergency department in March 2025 with complaints of dysphagia. Her medical history included asthma, a hiatal hernia complicated by oesophagitis and erythematous gastritis. She had recently completed eradication therapy for *Helicobacter pylori*, treated with amoxicillin and clarithromycin two weeks prior to presentation.

She first attended the emergency department for a rapidly progressive dysphagia evolving over 48 hours, initially limited to liquids and subsequently affecting solids as well. The initial clinical assessment was reassuring, with normal vital signs and unremarkable blood tests. A diagnosis of gastro-oesophageal reflux disease was made, and the patient was discharged with medical advice, a prescription for omeprazole 40 mg/day, and a referral for an outpatient upper gastrointestinal endoscopy.

Less than 24 hours later, the patient was readmitted due to clinical deterioration characterised by worsening dysphagia, dysarthria, dyspnoea, and weakness affecting all four limbs, predominantly proximally. Neurological examination was abnormal, revealing bilateral ptosis, binocular diplopia, dysarthria, dysphagia, and marked fatigability with difficulty walking. Motor testing showed proximal weakness of all four limbs, rated 3/5 on the Medical Research Council scale.

Venous blood gas analysis and routine blood tests were within normal limits. Serological testing for anti-acetylcholine receptor antibodies (AChR-Ab) and anti-muscle-specific kinase antibodies (MuSK-Ab) was requested in the emergency department but returned negative a few days later (Table 1). A non-contrast brain CT scan showed no acute abnormalities. Given the suspicion of a neuromuscular junction disorder, a neurological consultation and electromyography were requested, both supporting a diagnosis involving the neuromuscular junction (Table 2).

**Table 1:** Blood results and serologies during hospitalization *Older immunization: past Epstein-Barr Virus infection. EBV: Epstein-Barr virus, VCA: viral capsid antigen, CMV: cytomegalovirus, Ag: antigen, Ac: anticorps, ENA: extractable nuclear antigen, RNP: ribonucleoprotein, SSA: Sjögren's syndrome-related antigen A autoantibodies, SSB: Sjögren's syndrome-related antigen B autoantibodies.

Blood results	Normal values	Admission data
Anti-EBV VCA IgG (U/mL)	<20: Negative, ≥20: Positive	42, IgG positive, older immunization*
Anti-EBV VCA IgM (U/mL)	<20: Negative, 20-40: Equivocal, >40: Positive	<10.00
CMV IgG (U/mL)	<12: Negative, 12-14: Equivocal, >14: Positive	<5.00
CMV IgM (U/mL)	<18: Negative, 18-22: Equivocal, >22: Positive	8.51
HIV (Ag + Ac)	-	Negative
Herpes simplex (1+2) IgG (mUI/mL)	<0.9: Negative, 0.9-1.1: Equivocal, >1.1: Positive	2.6, positive
Herpes simplex (1+2) IgM (mUI/mL)	-	Positive
Varicella-Zoster virus (VZV) IgG (mUI/mL)	<150: Negative, ≥150: Positive	Negative
Varicella-Zoster virus (VZV) IgM (mUI/mL)	<0.9: Negative, 0.9-1.1: Equivocal, >1.1: Positive	Negative
Toxoplasmosis IgG (U/mL)	<7.2: Negative, 7.2-8.8: Equivocal, >8.8: Positive	62
Toxoplasmosis IgM (U/mL)	<6: Negative, 6-8: Equivocal, ≥8: Positive	Negative
Borrelia IgG (U/mL)	<10: Negative, 10-15: Equivocal, >15: Positive	<5.00
Borrelia IgM (U/mL)	<6: Negative, 6-8: Equivocal, >8: Positive	>15: Positive, patient immunized
Syphilis antibodies	-	Negative
ENA antibodies (RNP, SSA, SSB, SCL-70, JO1, Sm)	<0.7: Negative, 0.7-1: Equivocal, >1.0: Positive	0.30
Thyroid-stimulating hormone	0.35-4.94 mU/L	0.52
Free-T4	9.01-19.05 pmol/L	15.36
Free-T3	9.01-19.05 pmol/L	3.7
Anti-thyroglobulin antibodies	<4.11 UI/mL	1.17
Myositis antibodies	-	Negative
Anti-DNA Farr	-	Negative
Anti-acetylcholine receptor (AChR) antibodies	-	Negative
Anti-muscle-specific kinase (MuSK) antibodies	-	Negative

**Table 2:** Values obtained from repetitive nerve stimulation during electromyography (EMG) Electromyography performed in the emergency department on several nerve-muscle pairs (axillary nerve-deltoid muscle, accessory nerve-trapezius muscle, facial nerve-periorbital muscle, on both the left and right sides) demonstrated a significant decrement ranging from 5% to 18%, consistent with a neuromuscular junction disorder. The aim of this test is to assess changes in muscle response associated with pathological impairment of neuromuscular transmission, reflected by the presence of a decrement (expressed as a percentage). A decrement of ≥10% is considered significant and consistent with a neuromuscular junction disorder. Each nerve-muscle pair was stimulated repetitively in 1 to 4 series. Each series consisted of 10 stimuli delivered at a defined intensity (expressed in millivolts) and at a fixed frequency of 3 Hz. Series 3 and 4 were usually performed after exercise. Recordings were obtained using a stimulation electrode and a recording electrode positioned along the anatomical course of the nerve. A reference electrode was placed adjacent to the recording electrode. Ref.: Reference electrode, Rec.: Receptor electrode, Stim.: Stimulation electrode, mV: millivolts, ms: milliseconds.

	Series	Amplitude (mV)	Decrement amplitude 1 (%)	Decrement surface 1 (%)	Surface (mV/ms)
Left facial nerve: periorbital muscle, Ref. Nasalis, Rec. Nasalis, Stim. postauricular	1	1.79	-1.4	-3.8	4.8
	2	1.69	13.2	7.5	4.1
	3	1.82	-4.7	-2.9	4.2
	4	1.76	-13.6	-11.1	4.0
Right axillar nerve: deltoid muscle, Ref. subdeltoid, Rec. deltoid, Stim. suprasternal notch	3	4.50	-3.2	-8.1	21.9
	4	1.49	29.4	5	7.2
	5	4.54	-4.5	-5.4	21.3
Right facial nerve: periorbital muscle, Ref. Nasalis, Rec. Nasalis, Stim. postauricular	1	2.28	3	-18.2	8.0
	2	2.15	2.3	-7.3	7.7
	3	2.40	-3.6	-2.5	7.8
	4	2.36	0.4	-3.2	7.9
Right accessory nerve: trapezius muscle, Ref. Trapezius, Rec. Trapezius, Stim. Neck	1	3.12	-3	-8	15.8
	2	3.26	-1.5	-6.5	16.0

Due to a strong clinical suspicion of a de novo generalised myasthenic crisis with both limbs and bulbar involvement, a diagnostic neostigmine test, preceded by 0.25 mg of atropine and followed by 0.25 mg of neostigmine, was performed in the emergency department. The test was well tolerated haemodynamically but did not lead to any significant neurological improvement.

Given the absence of respiratory exhaustion upon initial evaluation in the emergency department, the patient was first admitted to the neurology ward. A nasogastric tube was placed to allow the administration of pyridostigmine, 30 mg three times daily. Unfortunately, her neurological condition deteriorated within hours of admission, leading to respiratory exhaustion and the onset of respiratory acidosis. She was subsequently transferred to the intensive care unit (ICU) for orotracheal intubation and mechanical ventilation.

The infectious serological workup (HIV, syphilis, Borrelia, herpes simplex virus, varicella-zoster virus, cytomegalovirus (CMV), Epstein-Barr virus (EBV), and toxoplasmosis) was non-contributory (Table 1). An inflammatory panel, including antinuclear antibodies (ANA) and anti-double-stranded DNA (dsDNA, Farr assay), also returned negative. Anti-acetylcholine receptor and anti-MuSK antibodies were negative, as were anti-GM1 and anti-GQ1b antibodies. Testing for thiopurine S-methyltransferase (TPMT) gene mutation was initiated in anticipation of introducing azathioprine as a long-term immunosuppressive therapy.

**Figure 1:**
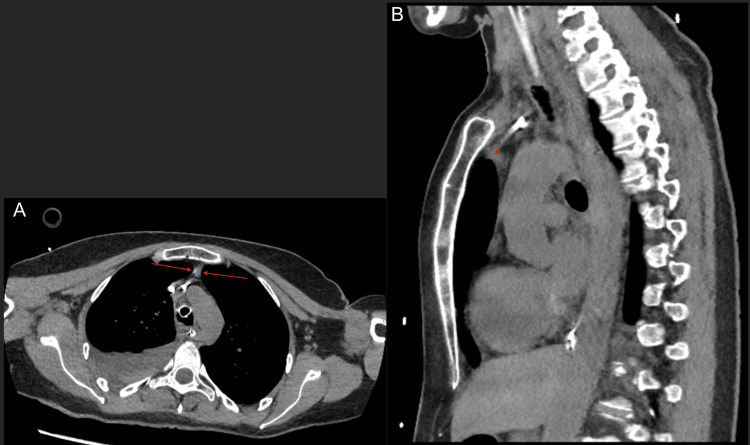
Thoracic CT scan showing the mediastinal space Left image (A) shows an axial view of the thoracic cavity with normal lung parenchyma, associated with moderate right pleural effusion and an anterior mediastinal space containing a small, non-specific ovoid thickening measuring 11 × 6 × 11 mm (red arrows; 30 Hounsfield units (HU)), possibly corresponding to residual thymic tissue or a thymic lesion. Right image (B) shows a sagittal view of the same thoracic cavity and the same non-specific ovoid thickening (red stars).

**Figure 2:**
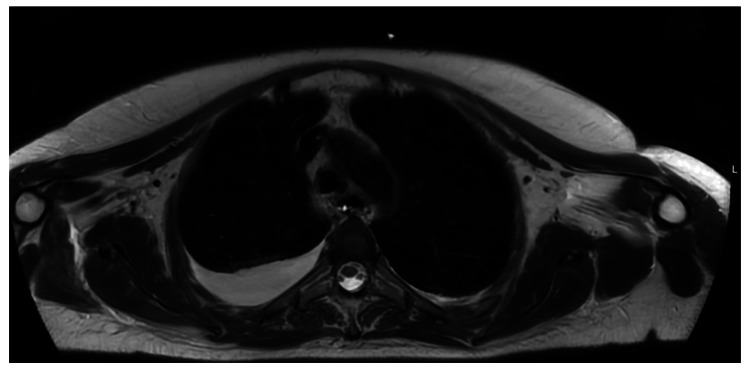
Magnetic resonance imaging (MRI) showing the mediastinal space on an axial view Residual thymic tissue in the anterosuperior mediastinum.

A chest CT scan performed 72 hours later revealed a mediastinal mass consistent with either residual thymic tissue or a possible thymoma (Figures 1A, 1B). A subsequent magnetic resonance imaging (MRI) scan conducted 15 days later confirmed the presence of an anterior-superior mediastinal remnant of thymic tissue, although it remained uncertain whether this represented a true thymoma (Figure 2). Further investigations are ongoing.

From a therapeutic perspective, the patient was treated with gradually increasing doses of pyridostigmine (30 mg three times daily) starting from her initial admission to the emergency department. As acute treatment, she underwent a total of five plasma exchange sessions, spaced 48 hours apart, followed by a course of intravenous immunoglobulin (IVIG) at a dose of 0.4 g/kg/day for five consecutive days. For long-term management, corticosteroid therapy with methylprednisolone was initiated and progressively increased, reaching a dose of 48 mg/day 10 days after ICU admission. Mycophenolic acid was also started at a dose of 500 mg twice daily, on day 13 of her ICU stay.

The clinical course was slow but favourable, with rapid improvement in limb strength and successful extubation after 16 days of mechanical ventilation. No infectious complications or ventilator-associated pneumonia occurred. The patient gradually resumed full oral feeding without any need for texture modification by the time of intensive care unit discharge. She was transferred back to the neurology ward 20 days after her admission to intensive care.

The final diagnosis retained was that of a de novo generalised seronegative myasthenia gravis, presenting with severe symptoms from the outset. The patient was discharged home 24 days after her initial admission to the emergency department, on maintenance therapy comprising methylprednisolone, mycophenolic acid, pyridostigmine, and pantoprazole. She showed an excellent recovery, with significant improvement in respiratory, swallowing, speech, and motor function. The patient’s neurological status showed significant improvement, with no major sequelae, allowing for substantial recovery of functional independence at home.

A follow-up electromyography performed 10 days after discharge, while the patient was asymptomatic under treatment, was normal with no decremental response. A subsequent outpatient MRI scan was unremarkable. Anti-acetylcholine receptor and anti-MuSK antibodies will be re-checked six months after the acute phase to assess for possible seroconversion.

**Figure 3:**
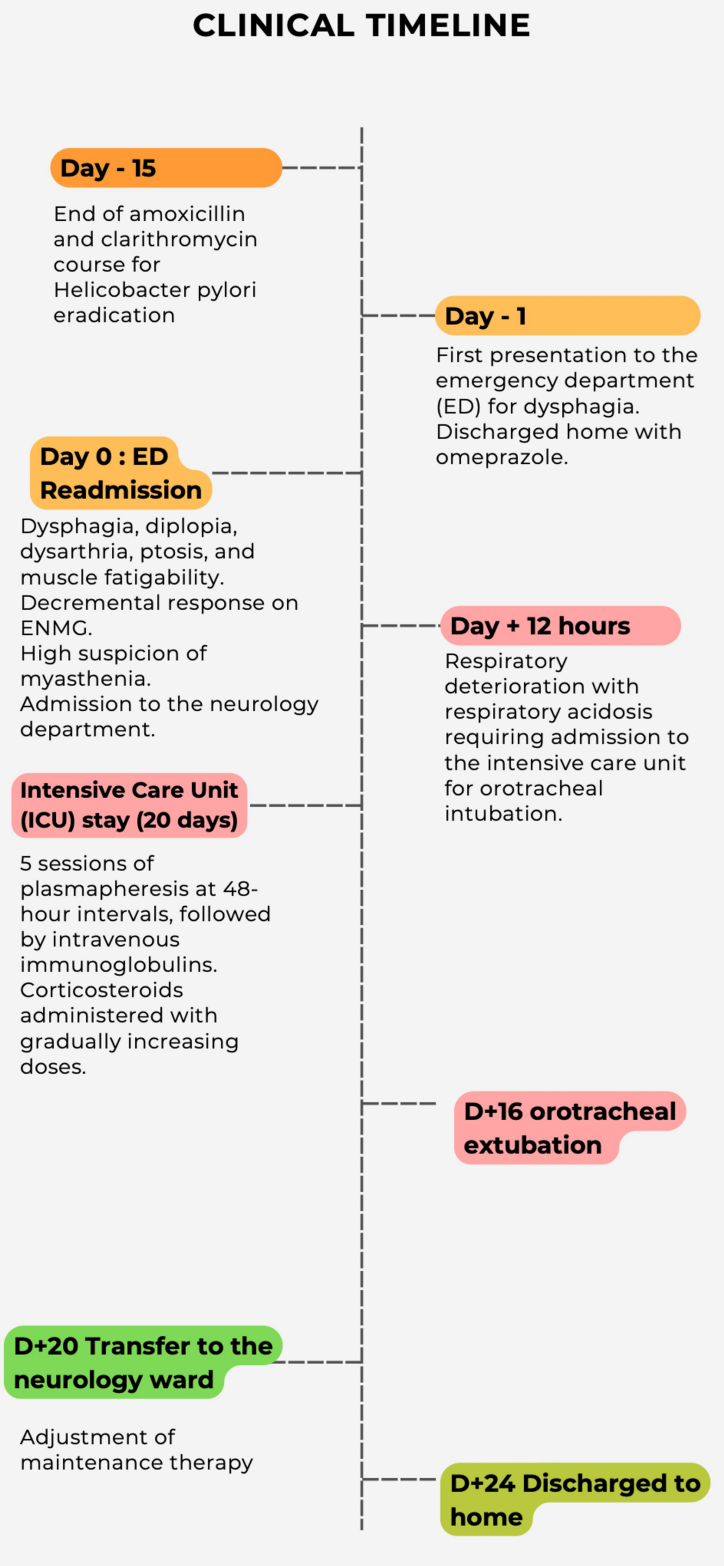
Clinical timeline ED: emergency department, ENMG: electroneuromyography, D: day.

The residual thymic tissue remains under evaluation. A positron emission tomography (PET) CT scan is underway, as there is currently limited evidence to support the presence of a thymoma. The indication for delayed surgical resection is still under consideration. A summary of the clinical presentation is provided in Figure 3.

## Discussion

We present here a case of newly diagnosed myasthenia gravis, atypical in its severity, as the initial dysphagia rapidly progressed to respiratory failure within 24 hours of admission to the emergency department. During the second emergency department admission, it was the combination of dysphagia with neurological deficits (fatigability syndrome), together with the EMG findings, that led to the diagnosis.

Serological investigations have thus far returned negative. The differential diagnosis included Guillain-Barré syndrome, as rare ascending bulbar variants have been described in the literature [10]. However, the rapid, generalised onset without an ascending pattern, preservation of deep tendon reflexes, and absence of axonal or demyelinating findings on electromyography are all strong arguments in favour of a myasthenic crisis [8,11]. Acute motor axonal neuropathy (AMAN) variants also exist, although these are more commonly reported in Asia [11]. Despite the absence of a lumbar puncture in this case, the rapid and favourable evolution without a “plateau” phase also supports the diagnosis of myasthenia rather than Guillain-Barré syndrome.

Myasthenia gravis results from an autoimmune attack on the postsynaptic acetylcholine receptors at the neuromuscular junction [7]. Approximately 20% of patients with myasthenia gravis may develop a severe myasthenic crisis [5]. Myasthenia gravis encompasses several pathogenic variants (early-onset, late-onset, thymoma-associated, seronegative, or ocular), each requiring distinct therapeutic approaches [7].

Although dysphagia reflects bulbar involvement in myasthenia, it is rarely the initial or isolated presenting symptom [5]. In myasthenic crises, bulbar impairment indicates severity due to the risk of aspiration, particularly when associated with respiratory muscle weakness leading to acute respiratory failure [5]. The main diagnostic challenge for clinicians lies in considering this rare disease in the context of a common and non-specific symptom such as dysphagia.

Tanaka et al. described three cases of isolated dysphagia revealing severe myasthenia, all requiring intubation and intensive care unit admission [1]. In other reports, dysphagia remained the sole manifestation for years, often misdiagnosed as peptic strictures, before progressing rapidly to myasthenic crisis, as illustrated by Klair et al. [2]. Sariaslani et al. reported a case of isolated dysphagia in a man in his 40s, diagnosed during speech therapy evaluation, with a favourable response to neostigmine and corticosteroids [3]. Abudalou et al. described a similar presentation in a 78-year-old man with anti-AChR antibody-positive myasthenia [4]. Finally, a recent Italian retrospective series of 80 patients, mostly with seronegative forms, highlighted both the diagnostic challenges, with 36% initially misdiagnosed, and the critical role of electromyography in diagnosis [12].

Macrolides, including clarithromycin, are among the antibiotics associated with a potential risk of triggering a myasthenic crisis, although the risk is considered low to intermediate [13]. However, more recent observational and cohort studies suggest that the absolute risk of exacerbation related to macrolide use is lower (<2.5% of usage episodes) and that the underlying infection is often the primary trigger [13,14]. In our case, recent *Helicobacter pylori* exposure and clarithromycin treatment may have contributed to the acute decompensation, despite a 15-day symptom-free interval prior to admission. However, in 30%-40% of myasthenic crisis cases, no obvious trigger can be identified [5,15]. The emergency management of a severe myasthenic crisis rests on three key pillars: maintenance of vital functions, identification and treatment of precipitating factors, and prompt administration of fast-acting immunomodulatory therapies [16].

The foremost priority is respiratory support. Intubation and mechanical ventilation should be considered early in cases of respiratory distress, although non-invasive ventilation may be trialled in selected stable patients, potentially reducing the duration of ventilation and length of stay in the intensive care unit [8]. Temporary discontinuation of acetylcholinesterase inhibitors is recommended during the acute phase, as they may worsen bulbar weakness and increase respiratory secretions [5,16,17]. Specific treatment involves the early administration of either plasma exchange or intravenous immunoglobulins (IVIG), which are considered equally effective for acute crisis according to international literature [18,19]. The choice between them depends on availability, patient profile, and contraindications. Plasma exchange appears to be associated with a lower likelihood of requiring invasive ventilation when used as first-line therapy [18,19]. High-dose corticosteroids are typically introduced early and gradually escalated, often after airway protection has been secured [5,8,16]. New immunomodulatory agents, such as complement inhibitors or neonatal Fc receptor blockers, are currently being integrated into the therapeutic arsenal, although their precise role in emergency settings remains to be determined [20]. The management of complications (such as pneumonia or sepsis) and a multidisciplinary approach in the intensive care unit are essential to improving patient outcomes [5,16].

## Conclusions

This case report highlights that isolated dysphagia, especially in patients with pre-existing gastrointestinal conditions, may mask an underlying neuromuscular junction disorder, leading to potential diagnostic delays. Myasthenic crisis can present atypically and progress rapidly, requiring a high index of suspicion in the emergency settings with atypical dysphagia with neurological signs. Early recognition and prompt respiratory support in severe myasthenic crisis, combined with immunomodulatory treatment such as plasma exchange or intravenous immunoglobulins, are crucial to improving outcomes. Macrolide antibiotics, including clarithromycin, may contribute to crisis exacerbation, but underlying infections remain the main precipitating factor.
